# Muscle and bone growth in the lower legs of typically developing children and ambulant children with cerebral palsy: a mixed longitudinal study

**DOI:** 10.1038/s41390-025-04558-0

**Published:** 2025-11-19

**Authors:** Bart Bolsterlee, Brian V. Y. Chow, Suzanne Davies, Catherine Morgan, Caroline D. Rae, David I. Warton, Iona Novak, Ann Lancaster, Gordana C. Popovic, Mahsa Seydi, Claudia Y. Rizzo, Iain K. Ball, Robert D. Herbert

**Affiliations:** 1 Neuroscience Research Australia (NeuRA), Sydney, NSW Australia; 2 Graduate School of Biomedical Engineering, University of New South Wales, Sydney, NSW Australia; 3 School of Biomedical Sciences, University of New South Wales, Sydney, NSW Australia; 4 Cerebral Palsy Alliance Research Institute, Discipline of Child and Adolescent Health, The University of Sydney, Sydney, NSW Australia; 5 School of Psychology, University of New South Wales, Sydney, NSW Australia; 6 School of Mathematics and Statistics, University of New South Wales, Sydney, NSW Australia; 7 Evolution & Ecology Research Centre, University of New South Wales, Sydney, NSW Australia; 8 Faculty of Medicine and Health, The University of Sydney, Sydney, NSW Australia; 9 Stats Central, Mark Wainwright Analytical Centre, University of New South Wales, Sydney, NSW Australia; 10 School of Population Health, University of New South Wales, Sydney, NSW Australia; 11 Philips Australia & New Zealand, North Ryde, NSW Australia

## Abstract

**Background:**

Longitudinal data on skeletal muscle growth in children are scarce, so little is known about developmental trajectories of skeletal muscle size, the relative timing of muscle and skeletal growth and how cerebral palsy (CP) affects growth.

**Methods:**

We conducted a mixed longitudinal study of 252 children aged 5–18 years, including 66 children with CP. MRI-based measurements of lower leg muscle volumes and tibia lengths were obtained on 2–3 occasions over ~3 years and used to estimate age- and sex-specific reference curves for growth velocities.

**Results:**

At the population level, peak adolescent growth velocity in lower leg muscle volume was higher and occurred later in typically developing boys (155 cm^3^/yr at 14.2 years) than in girls (117 cm^3^/yr at 12.1 years). In children with CP, median muscle growth and tibia length velocities were ~41% and 11% smaller, respectively, than in age- and sex-matched typically developing children. Muscle growth velocities in children with CP were low at all ages and did not systematically vary by degree of functional impairment, topography of CP or treatment history.

**Conclusion:**

This study establishes reference data for muscle and bone growth velocities during typical childhood development which can be used for child- and muscle-specific assessments of growth impairments.

**Impact:**

- Mixed longitudinal MRI data were used to construct reference curves for growth velocities of lower leg muscle volumes and tibia lengths in typically developing children aged 5–18 years.
- At the population level, peak adolescent muscle growth velocity was about one-third greater and occurred 2 years later in boys than in girls.
- Peak muscle growth velocities occurred 2 years after peak tibia length velocities, showing the asynchronous development of muscle and bone.
- Children with cerebral palsy exhibit larger deficits in growth velocities of muscles (~41% smaller than in age- and sex-matched typically developing children) than in tibia length (~11%).

## Introduction

Studies of musculoskeletal growth in children have largely served two purposes: to provide insights into the mechanisms or biology of growth,^1–3^ and to construct reference standards that can be used to distinguish normal from disordered growth.^4–6^ The present study aims to address both purposes by examining growth of skeletal muscle size and its relationship with longitudinal bone growth in typically developing (TD) children, and comparing muscle and bone growth between TD children and children with cerebral palsy (CP).

Growth is defined as change in size over time within an individual, so measurement of growth trajectories, or the derivative of size with respect to time—growth velocities - requires repeated measurements from the same individuals, i.e. longitudinal study designs. Ideally, measurements are obtained frequently from birth to adulthood. Ruff et al.^7^ analysed data from 10 boys and 10 girls whose calves were radiographed 29–38 times at ~6 month intervals from 6 months of age until late adolescence, creating a unique dataset.^8^ They found that growth trajectories of muscle widths differed substantially between individual children of the same age and sex, between muscles in the lower and upper limbs and between boys and girls. They also showed that peak growth velocities for muscles occurred later in adolescence than the peak for body height velocity, implying that muscles and bones develop asynchronously.

A more feasible alternative to the full longitudinal study design is the mixed cross-sectional and longitudinal study design (or mixed longitudinal design), in which repeated measurements are obtained over relatively short periods of time from children who enter the study at different ages. The measurements can be obtained at variable intervals. Tanner et al.,^3^ who popularised the mixed longitudinal study design for musculoskeletal growth research, measured calf and upper arm muscle widths 6-monthly from radiographs of the arms and legs of 280 boys and 252 girls aged 3–18 years at baseline. Children were followed up for 0–17 years (median of 3.5 years). This research confirmed the asynchronous timing of muscle and bone development and identified large between-child and sex differences in the onset of the pubertal growth spurt for muscle and bone. Xu et al.^1^ used computed tomography to measure muscle cross-sectional areas in a mixed longitudinal study on 258 pre-pubertal girls aged 10–13 years (four measurements over 7 years). They found a clear age-dependency of growth rates of muscle cross-sectional areas. The highest muscle growth velocities were observed ~1 year after peak tibia length velocity, qualitatively corroborating findings from the earlier radiography studies. This timing difference, however, contrasted with the findings from a longitudinal densitometry study, which found the growth velocity of lean body mass (a proxy for muscle mass) peaked ~0.7 years *before* the peak in bone mineral content accrual.^2^

Muscles have complex three-dimensional shapes, so measurements of muscle widths obtained from radiographs^3,7^ and measurements of muscle cross-sectional areas obtained from single computed tomography slices^1^ may not accurately represent volumetric growth.^9^ This limitation of one- and two-dimensional imaging methods is particularly important for longitudinal studies, because even though muscle thickness and cross-sectional area may correlate strongly with muscle volume at a particular time point, changes in thickness or area need not reflect changes in volume.^10^

To date, the most detailed longitudinal muscle growth studies have investigated the medial gastrocnemius in children, including children with cerebral palsy (CP). Cross-sectional studies^5,11^ have consistently reported that most children with CP have smaller muscles than their age- and sex-matched typically developing (TD) peers. Longitudinal studies have attempted to clarify when during development these differences arise. De Beukelaer et al.^12^ used 3D ultrasound to measure medial gastrocnemius volumes on 2–5 occasions over a 2 year period in 87 children with CP aged 6 months to 9 years at baseline. They found smaller muscle growth velocities in more functionally impaired children under 2 years of age, but no substantial differences in growth velocities by functional impairment in children aged 2–9 years. That study did not include a reference group of TD children. The same research group studied children at ~8 month intervals until 3 years of age^13^ and found that growth velocities in the medial gastrocnemius of 24 infants with CP were similar to those of 20 TD infants aged 0.5–1.5 years at baseline (mean growth velocities were ~12% smaller in children with CP, but this difference was not statistically significant). However, muscle volumes were ~40% smaller in infants with CP at 12 months of age. Another longitudinal study found reduced muscle growth velocities from 3 to 12 months of age in the triceps surae muscles of infants who graduated from neonatal intensity care units compared to TD infants, even when correcting for gestational age.^14^ These findings strongly suggest that calf muscles of children with CP (or at risk of developing CP) grow much more slowly than in TD children before the end of the first postnatal year. Because only one muscle has been studied in relatively small numbers of children up to 5 years of age, little is known about the growth of muscles other than the medial gastrocnemius, or about muscle growth after 5 years of age.

The current gold standard for muscle size measurements is magnetic resonance imaging (MRI).^15^ We have recently used MRI and artificial intelligence-enabled advances in automated segmentation^16^ to establish age- and sex-conditional reference curves for lower leg muscle volumes, based on cross-sectional data from 200 TD children aged 5–15 years.^5^ We demonstrated use of the curves to identify small-for-age muscles in children with CP. Here, we extend previous findings by analysing the mixed longitudinal data (two to three measurements over ~3 years) obtained from this cohort. Using a mixed longitudinal design, we aimed to determine age- and sex-specific distributions of lower leg muscle growth velocities in TD children. The data are used to examine the magnitude and timing of muscle and bone growth velocities during adolescence. We also use the data to compare muscle growth velocities of children with CP to those of TD children.

## Methods

### Participants

This study presents longitudinal data (two or three observations per child) from a cohort of children previously used for a cross-sectional study (one observation per child) on muscle growth.^5^ All 278 children (including 78 with CP) enrolled in the cross-sectional study were contacted for a second and third MRI scan with ~1.5 years in between scans. The cohort included children aged 5–15 years at study entry, who were recruited from the community and from CP clinics in New South Wales and Australian Capital Territory, Australia between 2019 and 2023. All scans were obtained between August 2019 and February 2025. Children were excluded from participation if they had a contraindication for magnetic resonance imaging (e.g. if they had ferrous implants), were unlikely to be able to remain still and relaxed during MRI scanning (e.g. because of dyskinesia, dystonia or behavioural reasons), or were known to have a condition (except for CP) that affects musculoskeletal growth. A parent or guardian of the participant provided consent and participants provided assent. All procedures conformed to the Declaration of Helsinki and were approved by the Sydney Children’s Hospital Network Human Research Ethics Committee (HC number: 2019/ETH11705). The full study protocol is available online.^17^

Body height was measured to the nearest millimetre while children were standing under a stadiometer. All children with CP in our cohort were ambulant so we could measure their height while standing. Body weight was measured to the nearest 0.1 kg using a digital scale with children wearing light clothing. For children with CP, their Gross Motor Function Classification (GMFCS) level and topography or CP (diplegic, hemiplegic or quadriplegic) was determined by an experienced paediatric neurological physiotherapist. We also recorded whether they had received botulinum toxin A injections below the knee, had lower limb corrective surgery, or wore an ankle-foot orthosis.

### Image acquisition and analysis

**Fig. 1:**
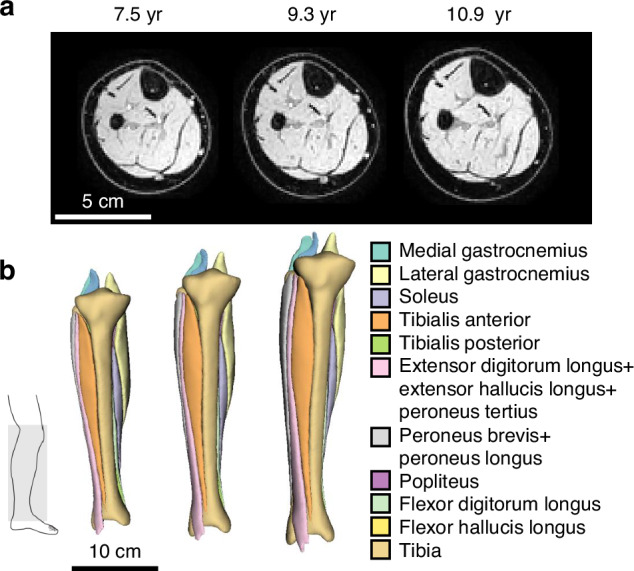
3D reconstruction of lower leg muscles and bones from MRI scans. **a** Example of transverse MRI slices (mDixon water image) obtained at approximately the same location midway between the ankle and the knee of a typically developing girl at age 7.5, 9.3 and 10.9 years. **b** Three-dimensional reconstructions of muscles and bones, obtained from segmentations of all slices of the scans. The surface models at the three time points are at the same scale, clearly showing longitudinal tibia growth from 27.7 cm to 33.6 cm over a 3.4 year period.

We used the same MRI acquisition protocol and image analysis procedures described previously.^5^ Briefly, children were positioned supine in a 3T Philips Ingenia CX MRI scanner (Philips Healthcare, Best, The Netherlands) using a combination of a dS torso/anterior coil and dS posterior coil with a combined number of 28 channels. mDixon MRI scans of the lower leg with a reconstructed image resolution of 1 × 1 × 1 mm were obtained with a variable number of slices between children to cover a volume spanning at least from the calcaneal insertion of the Achilles tendon to the origin of the gastrocnemius on the femoral condyle. We chose mDixon scans because of their fast acquisition, robustness to motion artefacts and better soft tissue contrast for segmentation of muscles than for example T1- or T2-weighted scans. Deep learning was used to segment 10 lower leg muscles or muscle groups (for muscles for which the boundaries could not be discerned clearly on the scans) and bones from the images, using an nnU-Net^18^ previously trained on segmented first scans of all children (Fig. 1). All segmentations were visually checked and corrected if needed. Muscle volumes were calculated as the product of voxel volume and the number of voxels in a muscle. Total lower leg muscle volume was the sum of the volumes of individual muscles. Tibia length was calculated as the length of a 3D surface model of the tibia along its first principal component.^19^

### Growth velocity curves for typically developing children

Growth velocities for body height, body weight, tibia length and lower leg muscle volumes were calculated by dividing the difference in value between subsequent measurements by the time between measurements. For this analysis, we assigned this velocity to the mean age between two measurements. For children with three measurements, two velocities were calculated. Semi-parametric quantile regression^20,21^ was used on growth velocities from TD children to estimate age- and sex-conditional distributions of growth velocities. The effect of age on growth velocities was modelled flexibly as a penalised b-spline with the smoothing term determined through an iterative procedure.^21^ Centile curves were estimated for boys and girls independently from the 1st to the 99th centile in steps of 1 centile. The 95% confidence bands around the curves were determined through block bootstrapping where, for each of 1000 bootstrap samples, children were sampled at random (with replacement) and all observations of selected children were included in the sample. In each bootstrap sample, the same random selection of children was used to estimate curves for body height, body weight, tibia length and muscle volumes to allow for paired (within-bootstrap sample) comparisons and timing of the adolescent growth spurt. Statistical analyses were conducted using R (version 4.4.3) and RStudio (version 2023.06.0).

### Analysis of the adolescent growth spurt

The highest growth velocities are typically found during the adolescent growth spurt. Because of the limited age range over which measurements were obtained in individual children, we could not determine peak growth velocity in individual children. However, we can determine the peak of the population median velocity (PPMV; i.e. the peak of the 50th centile curve) and the average age at which this peak occurred (age-at-PPMV; i.e. age at the peak of the 50th centile curve). The PPMV and age-at-PPMV were calculated for boys and girls separately. The 95% confidence intervals around the PPMV and age-at-PPMV were determined as the 2.5th and 97.5th centile value of the distribution of age-at-PPMV from 1000 bootstrap replicates. Differences between variables (body height, weight, tibia length and muscle volume) in age-at-PPMV were also calculated, and their 95% confidence intervals were calculated from the (paired) bootstrap samples. Because this analysis focuses on the adolescent growth spurt, we only considered peaks that occurred in the 50th centile curve above 8 and 10 years of age for girls and boys, respectively.

### Growth velocities in children with cerebral palsy

The reference curves for growth velocities were used to determine centiles for growth velocities of children with CP. The centile estimate for growth velocity for each variable of each child was obtained by finding the centile of the curve nearest to the observed velocity. A centile of, for example, 10 means that 10% of age- and sex-matched TD children have growth velocities smaller than the observed value. Growth velocities of children with CP were also expressed as a fraction of the age- and sex-matched 50^th^ centile growth velocities of TD children. A fractional velocity of, for example, 0.67 means that the growth velocity is two-thirds that of the median child of the same age and sex.

## Results

### Participants

**Fig. 2:**
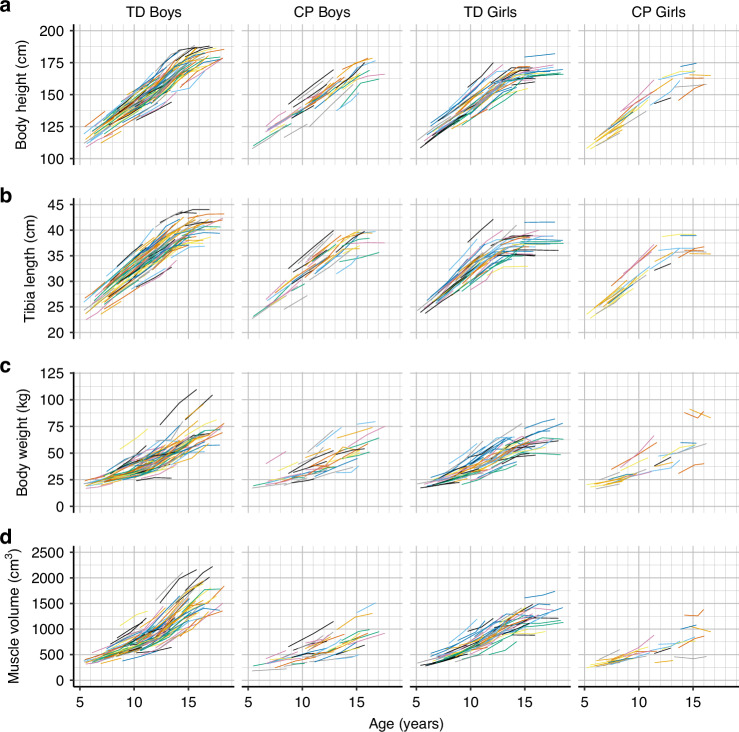
Longitudinal measurements for all study participants. Body height (**a**), tibia length (**b**), body weight (**c**) and total lower leg muscle volume (**d**) vs age for all participants. The lines connect observations on the same child. Children are given random colours which are kept constant across panels in the same column. From left to right, columns present data for typically developing (TD) boys, boys with cerebral palsy (CP), TD girls and girls with CP.

A total of 252 children were included in the present study as they were measured on at least two occasions (overall follow-up rate 91%; 186 TD children, follow-up rate 93%; 66 children with CP, follow-up rate 85%, follow-up rates are percentage of cross-sectional sample of 200 TD children and 78 children with CP^5^). A total of 218 children were measured on three occasions (overall follow-up rate 78%; 178 TD children, follow-up rate 89%; 50 children with CP, follow-up rate 64%). Children withdrew from the study because of relocation, loss of interest or inability to contact families. MRI data from one scan of one TD girl was excluded because of motion artefacts. Thus, we report 469 growth velocities from 252 children aged 5–18 years (Fig. 2). Table 1 summarises the cohort’s baseline characteristics. The mean ± standard deviation time between the first and last occasion was 3.0 ± 0.8 years (first to second: 1.7 ± 0.3 years; second to third: 1.5 ± 0.5 years).

**Table 1:** Baseline characteristics of participants.

	Typically developing		Cerebral palsy	
	Boys	Girls	Boys	Girls
# of participants measured on two occasions	108	78	40	26
# of participants measured on three occasions	98	70	30	20
Age at first scan (years)	10.0 ± 2.6	10.0 ± 2.7	10.2 ± 2.6	9.2 ± 3.1
Body height (cm)	142.0 ± 16.4	140.7 ± 16.5	139.3 ± 14.9	134.4 ± 20.2
Body weight (kg)	36.5 ± 12.8	36.0 ± 12.5	35.7 ± 13.4	35.1 ± 19.3
Time between 1st and 2nd scan (years)	1.7 ± 0.4	1.7 ± 0.2	1.7 ± 0.3	1.7 ± 0.2
Time between 2nd and 3rd scan (years)	1.5 ± 0.5	1.5 ± 0.6	1.3 ± 0.4	1.2 ± 0.5
GMFCS Level				
GMFCS Level I	—	—	28	14
GMFCS Level II	—	—	8	11
GMFCS Level III	—	—	4	1
Topography				
Left hemiplegia	—	—	9	9
Right hemiplegia	—	—	13	6
Diplegia	—	—	12	6
Quadriplegia	—	—	6	5
Has received botulinum toxin A injections below the knee?				
Yes			30	13
No			10	13
Has had lower limb surgery?				
Yes			9	3
No			31	23
Uses or has used an ankle-foot orthosis?				
Yes			25	18
No			15	8

Values are mean ± standard deviation (across children measured on at least two occasions) or number of participants within the category.

*GMFCS* Gross Motor Function Classification System.

### Growth velocity curves

**Fig. 3:**
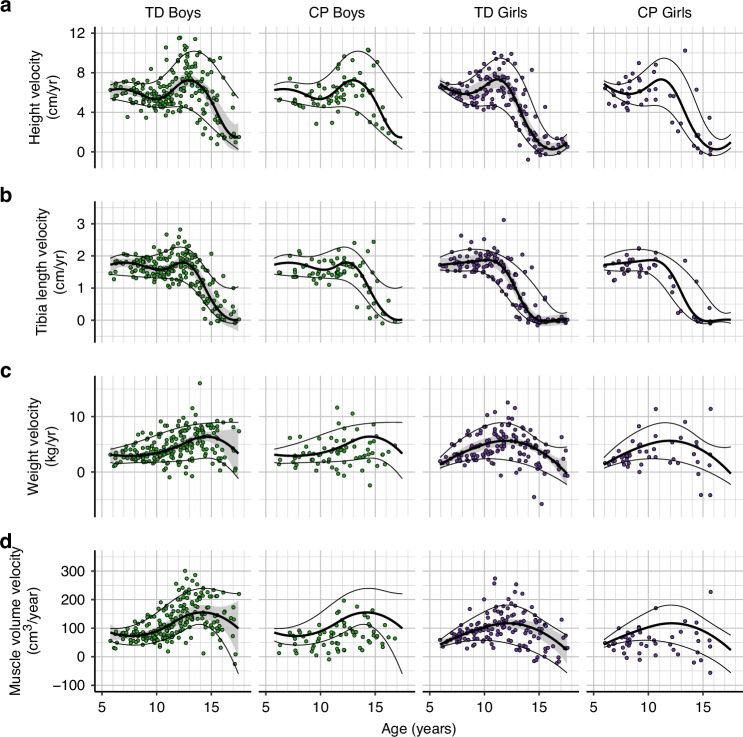
Reference curves for growth velocities. Reference curves for growth velocities in body height (**a**), tibia length (**b**), body weight (**c**) and total lower leg muscle volume (**d**). The curves were fitted on data from typically developing boys (column 1) and girls (column 3). The thick solid lines indicate the 50th centile curve and the upper and lower thin lines are the 10th and 90th centile curves, respectively. The grey shaded areas indicate the 95% confidence bands arounds the 50th centile curve. The same curves (but without confidence bands) are shown in column 2 and 4 with data points for boys and girls with cerebral palsy overlayed, respectively.

Growth velocities for all variables varied nonlinearly with age (Fig. 3). The shape of the median (50th centile) curves for growth velocity in body height and tibia length was similar for boys and girls. There was a period of relatively constant growth from 5 years of age until ~10 years (in girls) and 12 years (in boys). This was followed by a period of higher velocities during adolescence until the median boy and girl reached their final height and tibia length (growth velocities approaching zero) at approximately age 15 (girls) and 17 years (boys). Muscle volume and body weight velocities also showed relatively constant growth from 5 years of age until early adolescence, followed by higher growth velocities in adolescence. In the median boy, weight and muscle volume velocities remained high until the end of the age range studied here, whereas for the median girl these velocities approached zero by 18 years of age. Curves for individual muscles are presented in the Supplementary material (Figs. S1 and S2). In general, curves for individual muscles were similar in shape to curves for total lower leg volumes.

For all variables, the distributions of growth velocities were wide at any given age (see distance between the 10th and 90th centile curves in Fig. 3), but especially during the adolescent growth spurt. For instance, as a percentage of the peak of the 50th centile velocity curve, the 10th to 90th centile range for tibia length velocity was 17% for boys and 27% for girls at age 7, but 57% and 62% at age 13. Distributions for muscle volume velocities were even wider: 38% of the median peak velocity for boys and girls at age 7, and 81% for boys and 117% for girls at age 13.

Confidence bands around the 50th centile velocity curves were relatively narrow in the middle of the age range but wide at the ends of the age range where there were fewer observations (Fig. 3).

### Adolescent growth spurt

There was little difference between boys and girls in PPMV for body height, tibia length and weight velocity (Table 2). PPMV for muscle volumes was, however, substantially greater in boys than in girls (155 vs 117 cm^3^/yr).

**Table 2:** Peak population median velocities (PPMV) and age-at-PPMV for body height, tibia length, body weight and lower leg muscle volume for boys and girls.

	Boys		Girls	
	PPMV (/yr)	Age-at-PPMV (yr)	PPMV (/yr)	Age-at-PPMV (yr)
Body height velocity (cm/yr)	7.2 (6.7–7.9)	13.0 (12.6–13.6)	7.3 (6.2–8.0)	11.2 (10.5–11.5)
Tibia length velocity (cm/yr)	1.8 (1.6–1.9)	12.4 (12.0–12.9)	1.9 (1.7–2.1)	10.3 (8.4–10.8)
Weight velocity (kg/yr)	6.4 (5.4–8.1)	14.5 (13.8–17.4)	5.6 (5.0–6.4)	11.9 (11.2–12.8)
Muscle volume velocity (cm^3^/yr)	155 (138–186)	14.2 (13.3–15.6)	117 (102–128)	12.1 (11.3–13.1)

Values indicate the central estimate and 95% confidence intervals.

**Fig. 4:**
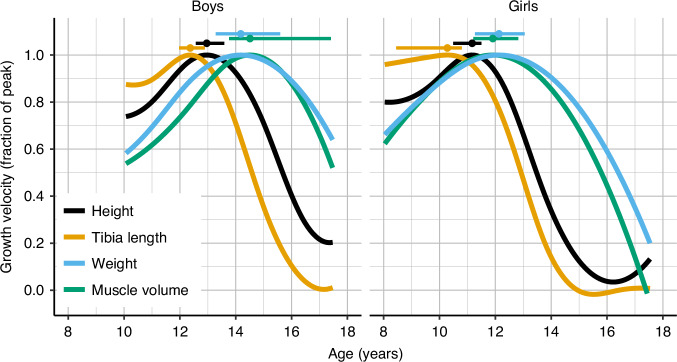
Timing of the adolescent growth spurt. The lines are the 50th centile curves for growth velocities in body height, tibia length, body weight and total lower leg muscle volumes for boys (left) and girls (right), normalised to the peak velocity. The points on top of the curves indicate the age of the peak of the curve (i.e. the age at which the population-median velocity peaks) and the horizontal bar indicates the 95% confidence interval around this age. Note that the curves boys and girls only show data from age 10 and 8, respectively, as this analysis focuses on the adolescent growth spurt.

The age-at-PPMV for muscle volumes occurred later in boys (at 14.2 years) than in girls (12.1 years). In boys and girls, the age-at-PPMV for muscle growth occurred after the age-at-PPMV for tibia length by 1.9 years (95% CI 1.0–3.7) in boys and 1.8 (95% CI 1.0–3.5) in girls (Table 2 and Fig. 4). The age-at-PPMV for tibia length occurred before age-at-PPMV of body height, by 0.7 years (95% CI 0.3–1.2 years) in boys and 0.9 (95% CI 0.4–2.6 years) in girls. The age-at-PPMV for muscle volume was not significantly different from age-at-PPMV for body weight (boys: 0.5 years (95% CI −0.8 to 3.3), girls: 0.0 (95% CI −0.8 to 1.1); positive values indicate higher age for body weight).

### Growth velocity in children with cerebral palsy

**Fig. 5:**
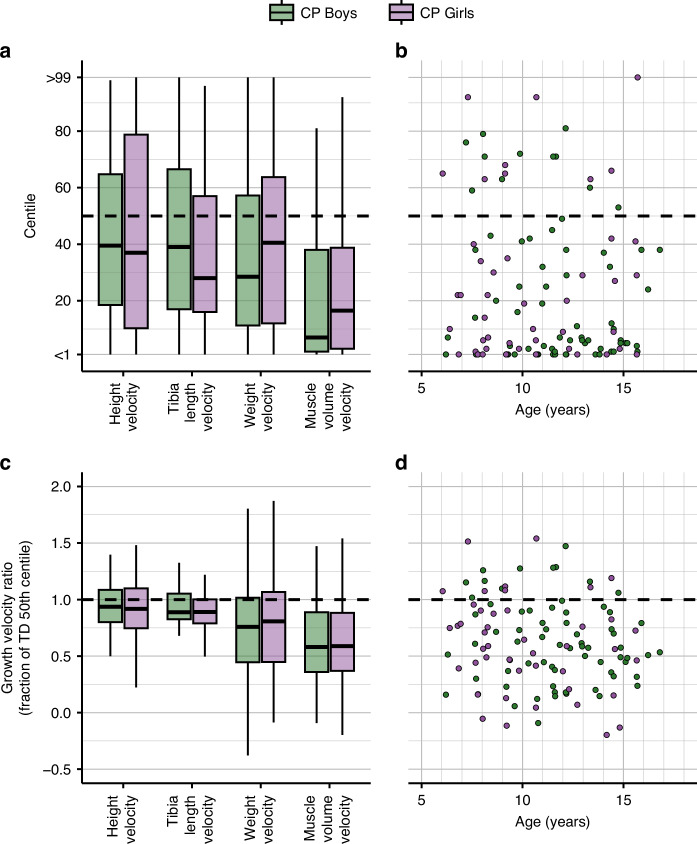
Centiles and growth velocity ratios for children with CP. **a** Boxplots of centiles of children with cerebral palsy (CP) for growth velocity in body height, weight, tibia length and total lower leg muscle volume. Boxes span the 1st to the 3rd quartile. Lower/upper whiskers indicate the minimum/maximum value of the data that is within 1.5 times the interquartile range under/over the 1st/3rd quartile. Median values are indicated by a thick black horizontal line. **b** Effect of age on centiles for muscle volume velocity for boys and girls with CP. In **a**, **b** the dashed horizontal black line at the 50th centile indicates the median value for typically developing (TD) children. **c** Boxplots of growth velocity ratios (i.e. fraction of the age- and sex-matched 50th centile value of TD children) of boys and girls with CP **d** Growth velocity ratios vs. age for boys and girls with CP. In **c**, **d** values below 1 (indicated by the thick horizontal black line) indicate smaller growth velocities in children with CP than in their age- and sex-matched TD peers.

Most boys and girls with CP had lower growth velocities in body height, tibia length, body weight and muscle volume than the median age- and sex-matched TD boy and girl (median centiles for boys and girls with CP < 50; Fig. 5a). Centiles were especially low for muscle growth velocities: median centiles for boys and girls with CP were 7 and 17, respectively. Muscle growth velocities in the median boy and girl with CP were 0.59 and 0.58 times that of the median age- and sex-matched TD boy and girl, respectively (Fig. 5c). Tibia length velocities were 0.9 and 0.89 times that of the median TD boys and girls. There was no clear effect of age on centiles or velocity reductions in muscle growth in children with CP (Fig. 5b, d).

**Fig. 6:**
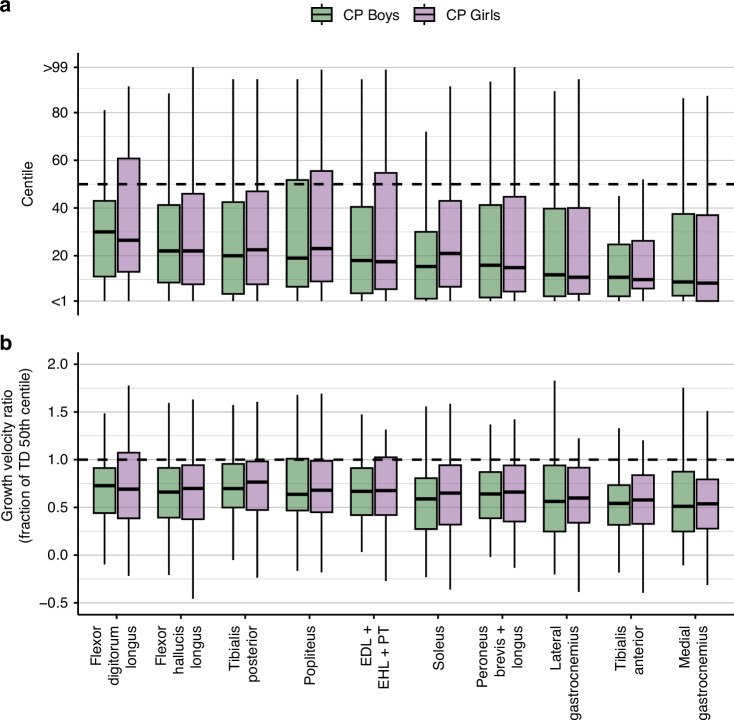
Centiles and and growth velocity ratios for individual lower leg muscles of children with CP. **a** Boxplots of centiles of children with cerebral palsy (CP) for growth velocity in 10 lower leg muscles or muscle groups. The muscles are sorted from left to right in decreasing median percentile. **b** Boxplots of growth velocity ratios (i.e. fraction of the age- and sex-matched 50th centile value of TD children) of boys and girls with CP for the same muscles as in (**a**). EDL extensor digitorum longus, EHL extensor hallucis longus, PT peroneus tertius.

Growth velocities for all individual muscles were low on the distributions of TD children’s growth velocities (centiles <50; Fig. 6a). The medial gastrocnemius had the lowest velocity ratio (0.51 for boys and 0.54 for girls; Fig. 6b). In girls, the tibialis posterior had the largest velocity ratio (0.75) and in boys the flexor digitorum longus (0.73).

## Discussion

Using mixed longitudinal MRI data of the lower legs of 252 children aged 5–18 years, we constructed reference curves for lower leg muscle growth velocities during typical childhood development. The curves show considerable between-child variation in age-specific growth velocities in TD children, sex differences in both magnitude and timing of peak muscle growth velocity during adolescence, and asynchronous growth of muscle and longitudinal bone growth. We also found that lower leg muscles in most children with CP grow substantially slower at all ages compared to their age- and sex-matched TD peers.

Distributions of muscle growth velocities were wide at all ages, in line with wide distributions of muscle volumes previously found in this cohort.^5^ Between-child variations in growth velocities in TD children were greater for muscle volume and body weight than for tibia length and body height, likely because skeletal growth is under tighter genetic control than muscle volume and body weight, which are influenced more strongly by environmental factors such as activity levels and nutrition.

At the population level, muscle volume growth in adolescence peaked 1.8–1.9 years later than peak growth in tibia length. The finding is qualitatively in accordance with previous studies on the timing of leg muscle and bone growth in adolescence.^1,3,7^ Ruff et al.,^7^ who measured growth trajectories of femoral muscle widths in a fully longitudinal cohort, found that age-at-peak-velocity in individual children averaged at 14.2 years for boys and 12.4 years for girls, close to the population-averaged values observed in our study (Table 2). This suggests that upper and lower legs follow similar growth trajectories. The finding that longitudinal bone growth precedes muscle growth raises the possibility that longitudinal bone growth is one of the factors controlling muscle volume growth in children, as chronic muscle stretch is known to increase muscle size in animals^22^ and in humans,^23^ although the mechanisms are poorly understood.^24^

Lower leg muscle volume velocity peaked around the same age as weight velocity. Muscle mass gain is likely a major contributor to weight gain in adolescence. Lower leg muscles constitute ~20% of total lower limb muscle mass,^25,26^ which in turn constitutes 57% of whole body muscle mass in women and 55% in men aged 18–29.^27^ Thus, the muscles in one lower leg constitute ~5.7% of total skeletal muscle mass in young women and ~5.5% in young men. Assuming a constant muscle density of 1.05 g/cm^3^.^28^ the peak growth velocity for lower leg muscle mass in boys is 0.163 kg/yr (=155 cm^3^/yr × 1.05 g/cm^3^; Table 2). If lower leg muscles remain an equal proportion of total muscle mass, muscle mass would accrue at 0.163/0.055 = 3.0 kg/yr in boys and at 2.2 kg/yr in girls at the peak of the adolescent growth spurt. Thus, by a first approximation, our data suggests that ~46% of the 6.4 kg/yr peak adolescent body weight gain in boys and ~38% of the 5.6 kg/yr in girls is due to increases in muscle mass.

Muscle growth velocities of most of the children in this cohort with CP were lower than in their age- and sex-matched TD peers. This was expected, as muscle volume is the time integral of muscle growth velocities and we^5^ and others^11,29,30^ had previously shown, in cross-sectional studies, that muscles of children with CP are typically small for age. This study provides two additional insights: muscle growth velocity is low-for -age across a wide age range (5–18 years), and growth of all lower leg muscles is impaired. The largest reductions in muscle volume growth velocity were found in the medial gastrocnemius muscle, for which the children with CP had 49% lower median velocities compared to TD children. The medial gastrocnemius is the only muscle that has been investigated in earlier longitudinal studies.^12,13,31^ The present study, combined with studies on children with CP younger than 5 years,^12,13,31^ show that muscle growth is impaired at all ages in children with CP. Future research should focus on why muscle growth is impaired across the age range, rather than trying to identify an age at which muscle growth becomes impaired.^29^

Muscle growth results from a complex interplay between genetic, hormonal and environmental factors, both before birth and during childhood, so the causes of reduced muscle growth in children with CP are difficult to identify. Moreover, many children with CP, including those in our cohort (Table 1), receive treatments such as botulinum toxin A injections or lower limb surgery which are likely to influence muscle growth. Further analysis of our dataset showed large overlaps in the distributions of growth velocities of children of varying GMFCS levels, topographies (hemiplegia, diplegia or quadriplegia) and treatment histories (Table 3). This demonstrates that growth impairments in children with CP cannot easily be explained by the degree of functional impairment, type of CP or treatment history alone and emphasises the need for individualised assessment of growth.

**Table 3:** Summary statistics for centiles for growth velocities in body height, tibia length, body weight and muscle volumes by sex, GMFCS Level and treatment history.

		Body height	Tibia length	Body weight	Muscle volume
Sex	Boy	40 (19, 65)	39 (17, 66)	29 (11, 57)	7 (2, 38)
	Girl	37 (10, 79)	28 (16, 57)	40 (12, 64)	16 (3, 39)
GMFCS	I	41 (18, 64)	38 (23, 64)	36 (12, 63)	13 (4, 40)
	II	30 (10, 78)	22 (8, 62)	38 (6, 63)	8 (1, 32)
	III	37 (14, 72)	37 (17, 71)	30 (17, 40)	4 (2, 21)
Topography	Diplegic	39 (16, 66)	40 (13, 58)	32 (7, 61)	15 (4, 36)
	Hemiplegic	40 (20, 64)	36 (22, 69)	38 (12, 64)	9 (3, 40)
	Quadriplegic	25 (8, 85)	17 (7, 53)	36 (13, 42)	7 (2, 41)
Botulinum toxin A^a^	Yes	43 (20, 77)	35 (16, 61)	36 (11, 56)	6 (2, 38)
	No	31 (9, 65)	36 (17, 68)	41 (12, 64)	19 (5, 42)
Lower limb surgery^b^	Yes	32 (16, 83)	42 (10, 79)	32 (11, 59)	6 (2, 31)
	No	40 (17, 66)	34 (16, 63)	36 (12, 60)	12 (3, 40)
Orthosis^c^	Yes	40 (17, 78)	38 (16, 68)	38 (11, 61)	7 (2, 34)
	No	37 (15, 58)	30 (18, 46)	22 (12, 58)	20 (4, 41)

The value in each cell represents the median (quartile 1, quartile 3) within the group.

*GMFCS* Gross Motor Function Classification Scale.

^a^Has the child had botulinum toxin A injections below the knee?

^b^Has the child had corrective surgery for the leg?

^c^Child uses or has used an ankle-foot orthosis?

The present study focused on muscle volumes, but muscles of children with CP also frequently differ from TD children’s muscles in their architecture,^32,33^ composition (e.g. they contain more fat^34,35^) and microstructure (e.g. they have more variable fibre diameters^36^). Another study found there were fewer muscle satellite cells in gracilis muscles of children with CP with hamstring contractures compared to TD children.^37,38^ Satellite cells play a fundamental role in muscle growth and regeneration following exercise or injury, so reductions in satellite cell numbers likely cause impaired growth. However, we do not know if reduced satellite cell numbers are a general feature of muscle in CP. Moreover, our data do not support the hypothesis that muscle growth ceases when the reserve of muscle satellite cells is completely depleted, because there was evidence of muscle growth in all children with CP regardless of age. Children with CP exhibited a growth spurt in adolescence. This observation suggests that muscle growth in children with CP can be accelerated by hormonal stimuli, just as it is in TD children. The presence of an adolescent muscle growth spurt in children with CP may also have implications for identifying the optimal timing of targeted interventions to stimulate muscle growth. Nevertheless, the causes of impaired muscle growth in children with CP remain unknown, which makes it difficult to develop effective interventions.

The statistical analysis of physical growth has a rich history,^6^ and advances in longitudinal analysis methods continue to be made.^39^ Growth trajectories differ strongly between individuals, so the most interesting insights are gained by observing growth trajectories in individual children over long periods. Individual growth trajectories can be fit accurately, at least for body height, from as few as four observations obtained 2 years apart using a nonlinear mixed modelling approach.^40,41^ For muscle volumes, which we showed are more variable across children, it is likely that more observations are required to model individual trajectories with sufficient accuracy. Unfortunately, we were only able to assess participants on up to three occasions over an average period of 3 years, and with variable measurement intervals. This poses limitations on the interpretation of our data. First, the peaks of the median population-averaged growth velocity curves do not generally correspond to the median peaks of the individual growth velocity curves.^3^ Each child’s peak occurs at a different age. As a consequence, the population-averaged peak growth velocities underestimate the average of the individual peak velocities. Second, we cannot estimate between-child variation in the magnitude or timing of the peak of the adolescent growth spurt, or the between-child variation in timing of the peaks for tibia length and muscle growth. Third, variable measurement intervals may have biased our growth velocities measurements, as errors are amplified more in measurements obtained over shorter time intervals. We do not think that this had a large impact on our findings, however, as the standard deviation of the time interval was relatively small (Table 1; 90% of measurement intervals were between 0.8 and 2.5 years). Moreover, visual inspection of growth velocities vs. measurement intervals did not reveal an obvious association between velocity and interval (data not shown). Finally, we only had few observations at the ends of the age range, so confidence bands were especially wide at older ages (see Fig. 3, Figs. S1 and S2). Thus, if the reference curves are to be used to identify impaired growth, they are best used for children aged 7–16 years who have an MRI-based measurement of muscle volume that is repeated within a 1–2 year interval.

In summary, this study shows how advances in automated analysis of MRI scans can be used for the study of muscle and bone growth velocities during typical childhood development, and for individualised assessment of impaired growth in children with CP. We have shown that, at the population-level, muscle growth velocities are about one-third greater and occur 2 years later in TD boys than in girls. In ambulant children with CP, muscle growth velocities are substantially reduced at all ages. The degree of impairment is not associated in any obvious way with functional impairment or treatment history, highlighting the need for individualised assessment of muscle and bone growth.

## Supplementary information

Supplementary information

## Data Availability

The datasets generated during and/or analysed during the current study are available for research purposes from the corresponding author on reasonable request.
