# Supplementary material for: Muscle and bone growth in the lower legs of typically developing children and ambulant children with cerebral palsy: a mixed longitudinal study
Source: Pediatr Res. 2025 Nov 19;99(7):2792–801. doi: 10.1038/s41390-025-04558-0 (PMC13485689; doi:10.1038/s41390-025-04558-0)
Supplement: Supplementary file 1 [file 41390_2025_4558_MOESM1_ESM.pdf]

Supplemental Information 1: Reference curve for individual muscles

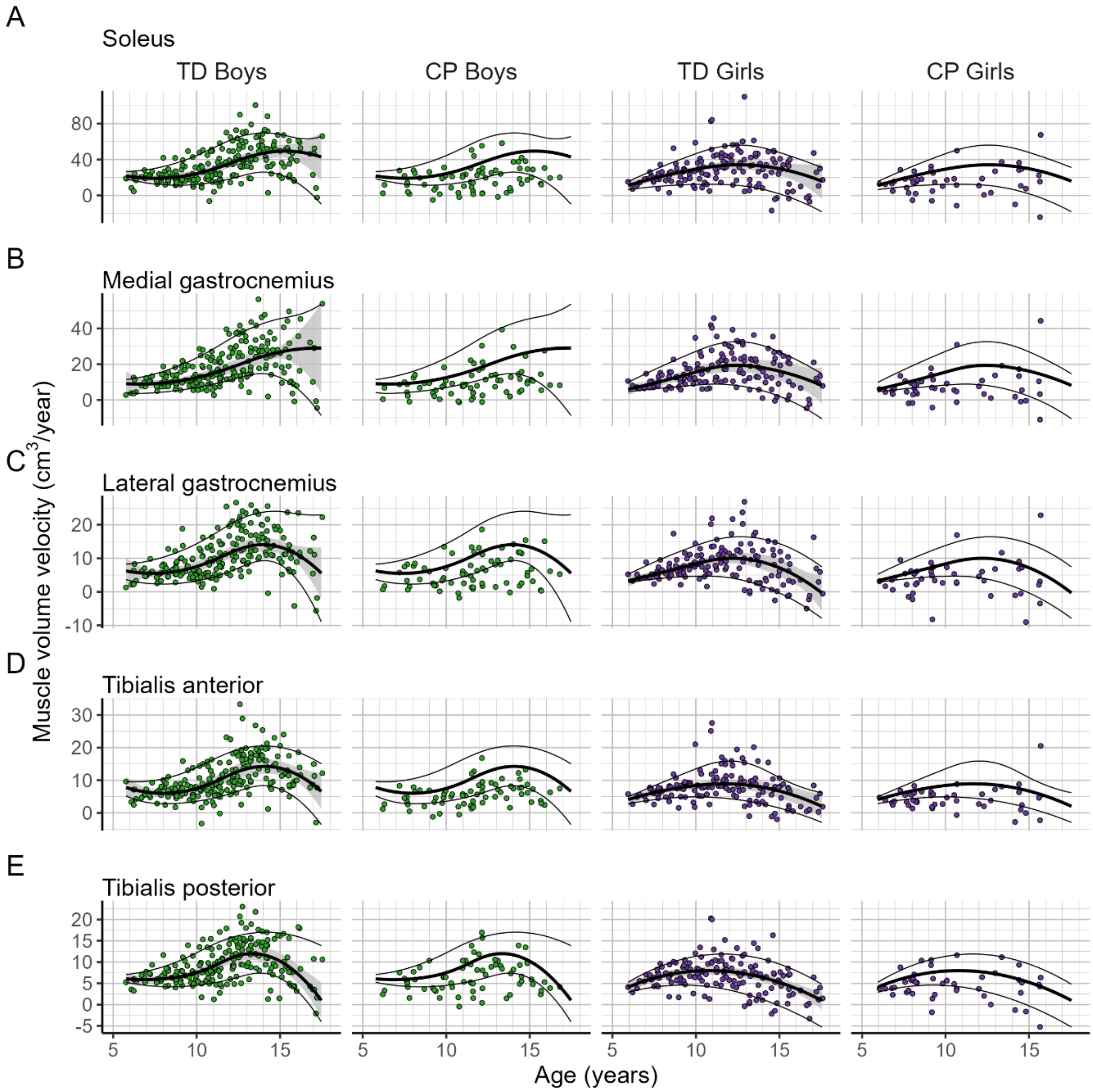

Fig. S1 Reference curves for growth velocities in muscle volumes of the A) soleus, B) medial gastrocnemius, C) lateral gastrocnemius, D) tibialis anterior and E) tibialis posterior. The curves were fitted on data from typically developing boys (column 1) and girls (column 3). The thick solid lines indicate the 50th centile curve and the upper and lower thin lines are the 10th and 90th centile curves, respectively. The gray shaded areas indicate the 95% confidence bands arounds the 50th centile curve. The same curves (but without confidence bands) are shown in column 2 and 4 with data points for boys and girls with cerebral palsy overlayed, respectively.

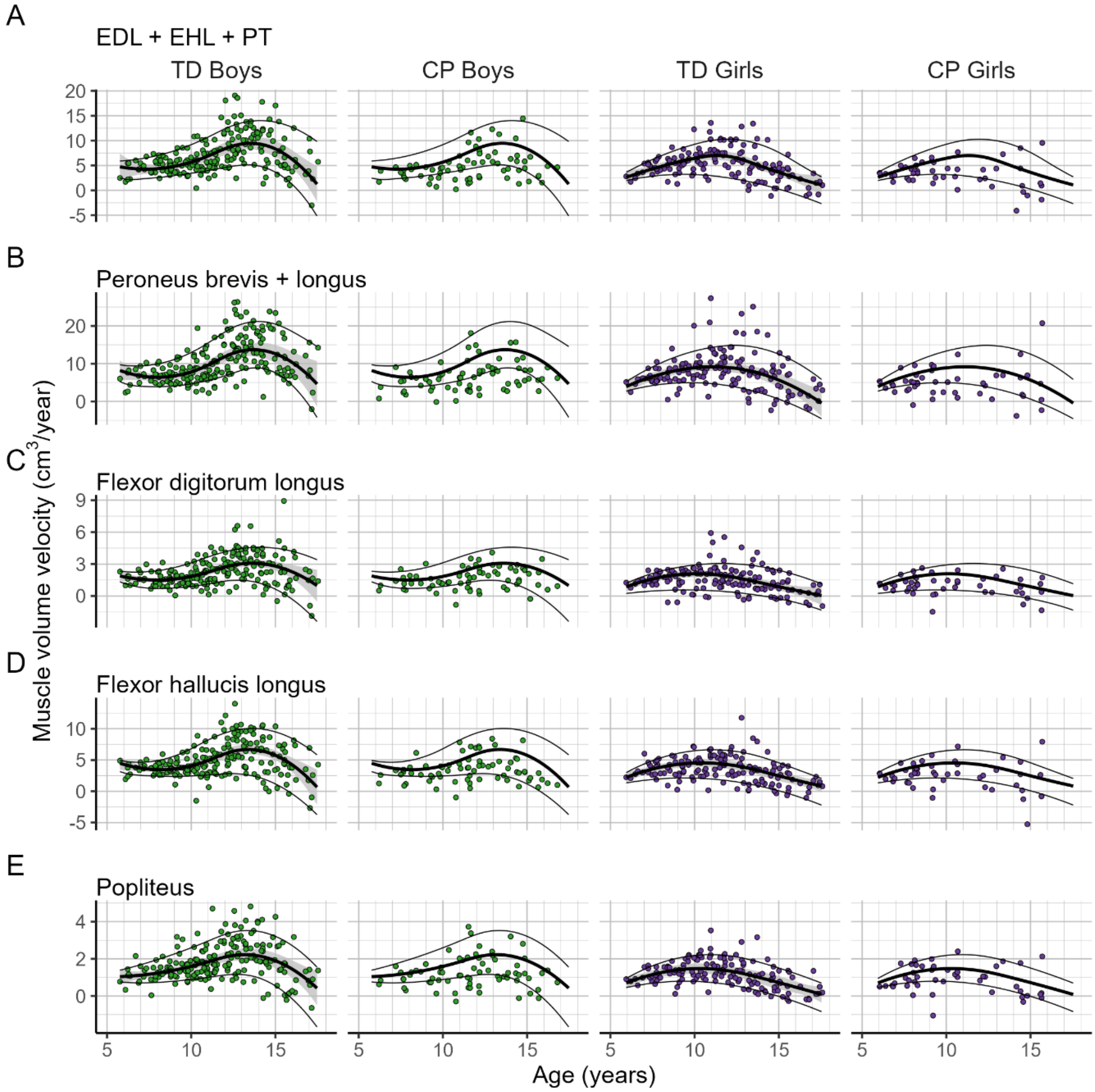

Fig. S2 Reference curves for growth velocities in muscle volumes of the A) combined volume of the extensor digitorum longus (EDL), extensor hallucis longus (EHL) and peroneus tertius (PT), B) combined volume of the peroneus brevis and peroneus longus, C) flexor digitorum longus, D) flexor hallucis longus and E) popliteus. The curves were fitted on data from typically developing boys (column 1) and girls (column 3). The thick solid lines indicate the 50th centile curve and the upper and lower thin lines are the 10th and 90th centile curves, respectively. The gray shaded areas indicate the 95% confidence bands arounds the 50th centile curve. The same curves (but without confidence bands) are shown in column 2 and 4 with data points for boys and girls with cerebral palsy overlayed, respectively.
